# ***Helicobacter pylori*** **and gastric cancer: a state of the art review**

**Published:** 2015

**Authors:** Sauid Ishaq, Lois Nunn

**Affiliations:** 1 *Gastroenterology department, Russells Hall Hospital, Birmingham City University, Birmingham, UK*; 2 *SGU Grenada, West Indies*

**Keywords:** *Helicobacter pylori*, Gastric cancer, Atrophic gastritis, Virulence, Pathogenesis

## Abstract

Gastric cancer is the third most common cause of cancer-related death in the world. It is now well- established that *Helicobacter pylori* infection predispose individuals toward gastric adenocarcinoma later in life. It has since been classified as a class I carcinogen by the World Health Organization. Research suggests that the oncogenic effects of *Helicobacter pylori* can occur through a variety of mechanisms, including the indirect inflammatory effects of *Helicobacter pylori* on the gastric mucosa and the direct epigenetic effects of *Helicobacter pylori* on individual cells. Whilst infected with *Helicobacter pylori*, a combination of environmental and host-dependent factors determines the likelihood of developing gastric cancer. Controversy remains regarding the effects of eradication of *Helicobacter pylori* on the prevention of further progression of gastric lesions and the possibility for regression of atrophic gastritis. The aim of this review is to synthesis different elements that contribute to the step-wise progression of normal gastric mucosa to gastric adenocarcinoma. This review helps clinicians to better identify those infected individuals who are at high risk of developing gastric cancer and implement the necessary investigations and treatment.

## Introduction

 Gastric cancer (*GC*) is the fifth most common cancer in the world and has the third highest mortality rates, for both sexes. In 2012, just fewer than 1,000,000 new cases of *GC* were diagnosed, and 723,000 deaths were attributable to it (1,2). *Helicobacter pylori* (*H. pylori*) plays a predominant role in the aetiology of *GC* and was characterised as a class I carcinogen by the World Health Organisation in 1994 (3). *H. pylori* is a microaerophilic gram-negative bacterium that colonises the gastric mucosa of 50% of the human population (4). The majority of infections are asymptomatic, therefore a screening and treatment program cannot be justified except for high-risk patients (5).

This review will assess the role of *H. pylori* in the pathogenesis of intestinal-type gastric carcinoma. The synergistic relationship of this bacterium with other host and environmental factors on the risk of subsequent neoplastic transformation will also be discussed. 

**Epidemiology of** ***H. pylori*** **and Gastric Cancer**

*H. pylori* infection rates vary across the world (6). However, there is little correlation between areas of high *H. pylori* infection rates and those with high prevalence of *GC* (6-8).African countries can see as high as 91% of their population infected with *H. pylori,* but have a very low prevalence of *GC* (8). A similar pattern has been reported in less developed countries in Asia such as India and Bangladesh. However, in more developed Asian countries such as Korea, Japan and China a positive correlation was reported between *H. pylori *infection rates and *GC* prevalence (9).

This variance may be explained by a combination of factors including: age at acquisition of infection, the type of *H. pylori* strains, the genetic profile of the host and environmental factors. 

**What is the pathogenesis of gastric cancer?**

There are many known risk factors for *GC* apart from *H. pylori* infection. A high salt or a low fibre diet, exposure to *N*-nitroso compounds from diet or smoking, alcohol consumption, low socioeconomic status, high BMI, old age and previous gastric surgery have all been linked to increased rates of *GC* (10). Sporadic genetic mutations are found more frequently than familial acquired mutations (97-99%) in intestinal-type *GC*, highlighting the role of environmental factors in this process (11). The *GC,* which appears in families are usually due to clustering of *H. pylori* infection. However, there are some rare conditions that increase the risk of *GC* including: hereditary non-polyposis colon cancer, Li-Fraumeni syndrome, Peutz-Jeghers syndrome, Familial Adenomatous Polyposis, Cowden syndrome, Lynch syndrome, pernicious anaemia and MUTYH-associated adenomatous polyposis (12). 

**How does** ***H. pylori*** **cause gastric cancer?**

There are two broad mechanisms in which *H. pylori* infection may eventually lead to intestinal-type *GC*.

***1) Indirect effects through inflammatory processes*** **.**

**Figure 1:**
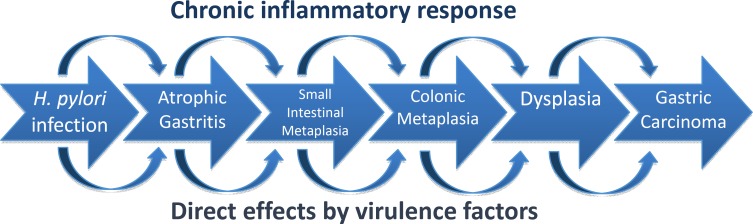
Correa’s hypothesis – the histopathologic stages from normal gastric mucosa to gastric carcinoma (14,15)

*H. pylori* infection mounts a chronic inflammatory response resulting in an increased cell turnover that, over several decades, may result in an accumulation of mitotic errors. The step-wise progression of this inflammatory process was illustrated by Correa (Figure 1) (13).

As illustrated above, persistent inflammation of the corpus by *H. pylori* results in atrophic gastritis, a risk factor for *GC* (16). Atrophic gastritis leads to a rise in pH and hypochlorhydria or achlorhydria. This alkaline environment facilitates the colonization and proliferation of *H. pylori *(17). Alternatively, with antral-predominant gastritis, hyperchlorhydria is observed resulting in duodenal ulcer disease, which confers a lower risk for developing *GC* (18-20). *H. pylori* infections initially cause antral gastritis, but in persistent infections, hypochlorhydria develops, allowing the bacteria to migrate proximally, resulting in pangastritis and an increased risk of adenocarcinoma. The clinical outcome depends on the interplay of the gastritis distribution and severity as well as the acid secretion (4).

*H. pylori*-gastritis is predominantly caused by a CD4 Th1-cell response (21). Neutrophils and macrophages are recruited which produce an excessive amount of reactive oxygen and reactive nitrogen species (ROS/RNS) (22-24). These reactive species, along with the superoxide and the hydroxyl ion produced by *H. pylori,* result in an increased oxidative stress and DNA damage (25).

Persistent infection by *H. pylori* is achieved through a variety of mechanisms. Firstly, *H. pylori* can protect itself from toxic substances such as oxidative species. Secondly, *H. pylori *can induce the apoptosis of macrophages and lastly, it can increase the expression of proinflammatory factors (26). Gastric mucosal expression of multiple cytokines (IL1B, IL-6, IL-8 and TNFα) and cyclooxygenase-2 (COX-2) is increased in the nucleus of the mucosal cells. This accelerates the progression of atrophic changes and induces intracellular signalling transformation (27-29). 

Aberrant DNA methylation in gastric epithelial cells occurs in parallel to the *H. pylori*-associated inflammatory response. This can have silencing effects on tumour suppressor genes (30).

***2) Direct effects***

*H. pylori* can have direct effects on the molecular make-up of the gastric epithelial cells through the toxic action of virulence factors. Mutations of cell-cycle regulating genes, deficiencies in DNA repair mechanisms, loss of a cell’s adhesive properties and epigenetic changes can alter the behaviour of the cell leading to cellular autonomy and malignant transformation. Studies in animals have shown an increased mutation rate in gastric mucosa infected with *H. pylori *(31).

Two extensively researched virulence factors are cytotoxin-associated gene A (CagA) contained with the Cag pathogenicity island (cagPAI) and vacuolating cytotoxin A (VacA). These virulent strains have been shown to be associated with precancerous gastric lesions and progression to a malignant phenotype (32,33). CagA positive strains have shown to produce a more potent inflammatory reaction causing a progression from gastritis to atrophy of the glandular mucosal cells and a higher risk of *GC*. CagA and peptidoglycan enter the epithelial cell through a bacterial type IV secretion system encoded by the cagPAI. CagA induces multiple cellular signalling pathways such as the mitogen-activated protein kinase (MAPK) cascade. Peptidoglycan induces NF-κB expression and phosphoinositide-3 kinase (PI3K-AKT) signalling pathways (34).

Additionally, cagPAI+ strains can directly induce gene mutations by enhancing the expression of the enzyme Activation Induced Deaminase (AID) in gastric mucosal cells. AID is a master regulator of secondary antibody diversification. AID causes mutations in the DNA encoding immunoglobulins. AID is exclusively expressed by B-lymphocytes, however *H. pylori* infection may lead to ectopic expression of AID and a high mutation rate of TP53 (35).

VacA contributes to the longevity of *H. pylori* infection by disrupting the epithelial cell barrier and suppressing the T-cell response. VacA strains that contain the alleles s1/m1 are particularly cytotoxic and produce their effect by inducing large intracellular acid vacuoles in the gastric epithelial cells. Additionally, VacA can upset the proliferation-apoptosis balance by activating proinflammatory intracellular signalling pathways and targeting the mitochondria, causing programmed cell death (36).

The prevalence of the two strains varies geographically and the carcinogenic risk imposed by *H. pylori* is determined by the strain-specific bacterial components. CagA and VacA s1/m1 strains have been found in Japanese and South Korean isolates where the prevalence of gastric cancer is high (37-39). The CagA+ strain is found less frequently in *H. pylori* isolates in India compared with Japan, where the incidence of *H. pylori* is similar yet incidence of *GC* is lower (40).

Other virulence factors, such as adhesions and outer membrane proteins including: BabA, DupA, FlaA, SabA and OiPA have been studied. E-cadherin is responsible for cell-cell adhesion in the gastric epithelium and has an important role in tumour suppression. Chan et al. reported E-cadherin hypermethylation was associated with an early *H. pylori* infection (41).

Pimental-Nunes et al. demonstrated a gradual increase in toll-like receptor (TLR) levels throughout the spectrum of gastric lesions listed in figure 1. This may suggest that TLRs play a role in the neoplastic transformation (42).

*GC* is one of the few solid cancers that involve large chromosomal aberrations such as fusion proteins. Toller et al. showed that gastric epithelial infection with *H. pylori* strain BabA lead to double stranded DNA breaks. Infection chronicity can result in genomic instability promoting the progression of gastric carcinogenesis (43). 

**What are the host factors that can affect the outcome of an H. pylori infection?**

There are naturally occurring genetic alterations that may not manifest themselves in a clinical syndrome or pathology, but instead increase the risk of *H. pylori*-related *GC*.

Such genetic alterations are single nucleotide polymorphisms (SNPs) found through genome wide association studies (GWAS). SNPs can alter gene expression and function. Prevalence of SNPs varies by ethnicity, which may be a plausible explanation for the discrepancy in host responses to *H. pylori* infection and subsequent disease outcomes among different regions of the world (44).

The most researched SNPs involve the proinflammatory cytokines: the interleukin-1 (IL-1) gene cluster, IL-10 and tumour necrosis factor-alpha (TNF-α) (45). These SNPs have been found to be associated with an increased risk of *H. pylori*-related non-cardia gastric adenocarcinoma. IL-1B is a potent inhibitor of acid secretion (46-48). Excess risk is observed in hosts with an SNP in the IL-1B gene as well as infection with *H. pylori *VacAs1/m1 and CagA+ (49). SNPs of IL-2, IL-4, IL-6, IL-12, IL-13 are all negatively associated with *H. pylori* infection (45).

**What are the environmental factors that can affect the outcome of an** ***H. pylori*** **infection?**

Environmental factors may have an additive effect on the *H. pylori*-associated risk of developing *GC*. 

The effect of diet has been extensively investigated. Dietary salt and *H. pylori* act synergistically: salt damages the gastric mucosa allowing *H. pylori* infection and persistence, increasing the susceptibility to tumorigenesis (50).

A diet high in pickled foods has been shown to increase the risk of *H. pylori*-associated *GC*, whilst the risk was lower in people with a high fibre intake (51). It is controversial whether red meat confers any excess risk in *H. pylori* infected individuals. It is well-known that *H. pylori* requires iron for growth and red meat has been shown to increase the risk of gastric adenocarcinoma in the absence of *H. pylori* through haem iron catalyzing the endogenous formation of carcinogenic *N*-nitroso compounds (52,53).

Poor glycaemic control has been found to increase the risk of *GC* in the setting of *H. pylori*. Ikeda et al. followed 2603 individuals with various HbA1c levels after 14 years and reported a greater risk of *GC* in those with high (>6.0%) HbA1c levels and a co-existent *H. pylori* infection (54).

It is evident that other gastrointestinal microbes act synergistically with *H. pylori* in the neoplastic process. Li et al. have demonstrated how antibiotic treatment for *H. pylori* significantly reduced the incidence of gastric cancer and mortality (55). Wong et al. have shown similar significant decreases in incidence of *GC* in patients who had already developed intestinal metaplasia and dysplasia. Interestingly, if the *H. pylori* infection persisted after eradication therapy, the incidence of *GC* was not significantly reduced, which may suggest *H. pylori* plays a role in the later stages of carcinogenesis as well as the early inflammatory process (56). Eradication of an *H. pylori* infection in individuals on long term proton pump inhibitor (PPI) therapy has been suggested. The rise in pH caused by PPIs can accelerate the progression of *H. pylori* gastritis to corpus atrophy. Whether this equates to an increased risk of carcinoma is yet to be established (57).

Wu et al. performed a retrospective study that demonstrated non-steroidal anti-inflammatories (NSAIDs) decrease the risk of *GC* in people with a history of *H. pylori* infection. NSAIDs may elicit a protective effect by decreasing the production of the pro-inflammatory molecule COX-2, which would normally be up-regulated by *H. pylori* infection (58).

Dietary intake of the antioxidants, vitamins C, E and beta-carotene may also be protective, and reduce the oxidative damage that occurs in early infection (59). Treatment of the infection plus supplementation showed a regression of preneoplastic lesions at a 6-year follow-up (60).

Helminth coinfection has also been demonstrated to reduce the severity of gastritis and progression to gastric atrophy (61,62).

**Can eradication reverse the damage done by H. pylori and does it decrease the risk of gastric cancer?**

A recent meta-analysis of six randomized controlled trials has shown eradication of *H. pylori* to reduce the *GC* incidence by 44%. This equates to 124 *H. pylori* infections need to be treated to prevent one case of *GC* (63). Similarly, in a 15-year follow up of a placebo-controlled trial, Ma et al. observed a 39% reduction in incidence of precancerous lesions after eradication (64). Additionally, Vannella et al. found that, eight years after eradication, 50% of patients treated for *H. pylori* had a reversal of atrophic body gastritis (65).

However, Fuccio et al. reviewed the available evidence and concluded that no study demonstrated a significant decrease in the incidence of *GC* with eradication therapy if the damage had exceeded atrophic gastritis. On the other hand, Fuccio et al. concluded that eradication could prevent the progression of preneoplastic lesions (66). This may suggest that infection eradication during the earlier stages of *H. pylori* infection eradication might be one of clinical benefits, however later there may be a ‘point-of-no-return’ where cancer risk is only reduced rather than fully eliminated. Li et al. challenged the concept of the ‘point-of-no-return’. Li et al. suggests that any age group, with any histopathological stage as late on as dysplasia could benefit from eradication therapy (55).

Eradication therapy does not always prevent *GC* or allow for regression of preneoplastic lesions (67,68). Even after eradication of *H. pylori* infection, extensive atrophic gastritis may remain, which is a significant risk factor for metachronous *GC*. Predictive markers such as pepsinogen I can be used to assess the future risk of cancer and surveillance endoscopy has an important role in monitoring those at high risk (69). 

## Conclusion

The aetiology of *GC* is complex and multifactorial, involving environmental and host related factors as well as genetic and epigenetic alterations. *H. pylori* infection is a necessary but not a sufficient cause for gastric cancer; hence current guidelines recommend population screening and treatment for *H. pylori* only in high-risk populations (70). 

Additionally, changes in lifestyle and dietary habits could further reduce incidence of *GC*. Prevention strategies should include identifying high-risk patients, targeting precursor lesions to offer a personalised therapy.

There is an urgent need for future research to focus on the genetic changes that are occurring in parallel to the inflammatory process to improve the outcomes of this killer disease.
